# Tumor Growth Decreases NK and B Cells as well as Common Lymphoid Progenitor

**DOI:** 10.1371/journal.pone.0003180

**Published:** 2008-09-11

**Authors:** John Richards, Beth McNally, Xianfeng Fang, Michael A. Caligiuri, Pan Zheng, Yang Liu

**Affiliations:** 1 Division of Cancer Immunology, Department of Pathology, The Ohio State University Medical Center and Comprehensive Cancer Center, Columbus, Ohio, United States of America; 2 Division of Hematology and Oncology, Department of Medicine, The Ohio State University Medical Center and Comprehensive Cancer Center, Columbus, Ohio, United States of America; 3 Division of Immunotherapy, Departments of Surgery, Internal Medicine, Pathology and Comprehensive Cancer Center, University of Michigan, Ann Arbor, Michigan, United States of America; 4 Institute of Biophysics, Chinese Academy of Science, Beijing, China; Centre de Recherche Public-Santé, Luxembourg

## Abstract

**Background:**

It is well established that chronic tumor growth results in functional inactivation of T cells and NK cells. It is less clear, however, whether lymphopoeisis is affected by tumor growth.

**Principal Findings:**

In our efforts of analyzing the impact of tumor growth on NK cell development, we observed a major reduction of NK cell numbers in mice bearing multiple lineages of tumor cells. The decrease in NK cell numbers was not due to increased apoptosis or decreased proliferation in the NK compartment. In addition, transgenic expression of IL-15 also failed to rescue the defective production of NK cells. Our systematic characterization of lymphopoeisis in tumor-bearing mice indicated that the number of the common lymphoid progenitor was significantly reduced in tumor-bearing mice.

The number of B cells also decreased substantially in tumor bearing mice.

**Conclusions and Significance:**

Our data reveal a novel mechanism for tumor evasion of host immunity and suggest a new interpretation for the altered myeloid and lymphoid ratio in tumor bearing hosts.

## Introduction

Hematopoiesis is the process that generates leukocytes, erythrocytes and megakaryocytes. It has been divided into two branches[1], the lymphoid branch that generates B cells, T cells, NK cells and dendritic cells, and the myeloid branch that produces granulocytes, monocytes, dendritc cells, erythrocytes and megakaryocytes [2]. There appears to be two layers of regulation [3]. The first layer of control has been refered to as basal hematopoiesis. It is responsible for maintaining normal blood cell production and is regulated by cytokines produced within the microenvironment of the bone marrow. The second regulatory layer has been called amplified hematopoiesis. Amplified hematopoiesis is caused by physiological stress and appears to be regulated by the endocrine system. Cytokines or metabolites produced beyond the microenvironment of the bone marrow signal the marrow by a combination of diffusion and plasma transport. An example of amplified hematopoiesis is found in tumor bearing mice. Many transplantable tumors secrete different cytokines that act on bone marrow precursors among which are GM-CSF, IL-3, M-CSF, IL-6 and VEGF [4]. In response to these cytokines there is an expansion of myeloid cells that lead to immunosuppression [5].

We have recently reported that natural killer (NK) cells are also affected by soluble factors that are associated with tumor growth [6]. In the aforementioned studies, NK cells were characterized as being CD11b^lo^ and had impaired function in vivo. The immature phenotype was associated with a deceased number of IL15Rα^+^NK1.1^−^CD3^−^ cells in the bone marrow. Inhibition of NK cell development has been blocked by the intentional disruption of several genes including Flt-3L [7] and IL15 [8]. In Flt-3L and interleukin (IL)-15 null mice, the NK cell number is drastically reduced [7], [8]. Depleted numbers of NK cells in Flt3L-deficient mice is due to fewer common lymphoid progenitors [9]. In vitro culture systems have demonstrated that Flt3L enhances the expression of CD122 on human hematopoietic stem cells [10]. In a two-step culture system used to generate NK cells from murine bone marrow, Flt3L is used in the initial step to produce IL-15 responsive cells [11]. Although kit ligand (KL) is also used in these cultures and stimulates CD122 expression, mice deficient in KL have normal numbers of NK cells in the periphery [12]. Disruption of IL-15 [8] or components of the IL-15 receptor[13], [14] reduce NK cell numbers by preventing the induction of Bcl-2 [15], [16]. Thus IL-15 acts as a survival factor in NK cell development.

In patients with CML, there is a progressive decrease in the number of NK cells [17]. We previously demonstrated that NK cell differentiation is inhibited in the bone marrow[6] and now hypothesize that NK cell numbers may be reduced in association with modifications of hematopoiesis. We found that as tumors progress NK cell numbers decrease. The reduced number of NK cells is not associated with an increase in apoptosis or a decrease in cellular proliferation, but a reduction in progenitor cell production. The decrease in the common lymphoid progenitor may be one early step of many which results in a dramatic decrease in lymphocyte production as there is a substantial decrease also found in B cell development.

## Methods

### Mice and Tumors

C57Bl/6 and Balb/c mice were purchased from Charles River Laboratories under contract from the National Cancer Institute. B6 PLThy1<a>/cy and C57Bl/6-LySU-Pep3B were purchased from the Jackson Laboratory (Bar Harbor, ME). IL-15Tg mice have been described [18]. All mice were housed in the University Laboratory Animal Facility at The Ohio State University and University of Michigan under specific pathogen-free conditions. All experiments utilizing animals were approved by an institutional review board.

Thymoma EL4, melanoma B16F1 and colon cancer cell line MC38 are syngeneic to C57Bl/6 mice. EL4 was grown in RPMI 1640 medium supplemented with 5% FBS, 100 U/ml penicillin, 100 µg/ml streptomycin and 4 mM L-glutamine. B16F1 and MC38 were all grown in DMEM medium supplemented with 5% FBS, 100 U/ml penicillin, 100 ug/ml streptomycin and 4 mM L-glutamine.

### Antibodies

The following fluorochrome-conjugated antibodies were purchased from eBioscience (San Diego, CA): Fluorescein isothiocyanate (Fitc)-conjugated anti-Sca1 (clone D7); allophycocyanin (APC)-conjugated anti-CD3ε (clone 145-2C11); APC anti-CD117 (clone 2B8); phycoerythrin (PE) anti-CD127 (clone A7R34) and PE anti-GR1 (clone RB6-8C5). The following fluorochrome-conjugated antibodies were purchased from BD Pharmingen (San Diego, CA): Peridinin chlorophyll protein Cy5.5 (PercpCy5.5)-conjugated anti-NK1.1 (clone PK136); PercpCy5.5 anti-B220 (clone RA3-6B2); Fitc anti-CD11b (clone M1/70); PE-anti-CD45.1 (clone A20); PE anti-CD122 (clone TM-β1); Fitc anti-CD49b/Pan-NK cells (clone DX5), Fitc anti-BrdU (clone 3D4); Fitc IgG1 isotype control (clone MOPC-21); PE-Annexin V and streptavidin-PercpCy5.5. The Mouse Lineage Panel consisting of biotinylated CD3, B220, CD11b, GR-1 and Ter119 were purchased from BD Pharmingen.

### Establishment of Subcutaneous Tumor

Syngeneic mice were subcutaneously injected with EL4 (5×10^6^ cells), B16F1 (1×10^4^ cells), or MC38 (5×10^5^ cells) viable tumor cells. Mice were monitored every 2–3 days to evaluate tumor growth and subcutaneous tumors were measured with a caliper along perpendicular axes of the tumor. Mice were sacrificed when tumors reached a size of 20 mm^2^.

### Cell Preparation and Flow Cytometry

Single cell suspensions were prepared from bone marrow and spleen and were depleted of red blood cells. For flow cytometry, cells were initially incubated with 2.4G2 supernatant to block nonspecific antibody binding. Cells were then stained with four-color combinations of indicated fluorochrome-conjugated monoclonal antibodies. Stained cells were fixed and analyzed with a FACsCalibur (Becton Dickenson, San Jose, CA).

### Apoptosis

Single cell suspensions were surface stained as described above, in combination with fluorochrome-conjugated Annexin V and samples were analyzed immediately with a FACsCalibur.

### Bone Marrow Transplant

C57Bl/6 mice were sublethally irradiated and injected with either EL4 tumors or PBS. 24 hours later bone marrow was obtained from 10 C57BL/6-LySU-Pep3B mice that had been treated 5-days earlier with 5FU. Approximately 1×10^6^ bone marrow cells were intravenously injected into C57Bl/6j mice that were previously injected with either PBS or EL4. After 3 weeks, cells were isolated from the spleen and bone marrow of recipient mice and analyzed by flow cytometry.

### Statistics

Unless otherwise stated statistics were calculated by using an unpaired Student's T test (two tails). Significance was designated as a p-value≤0.05.

## Results

### Tumor-associacted reduction of NK cell number

**Figure 1:**
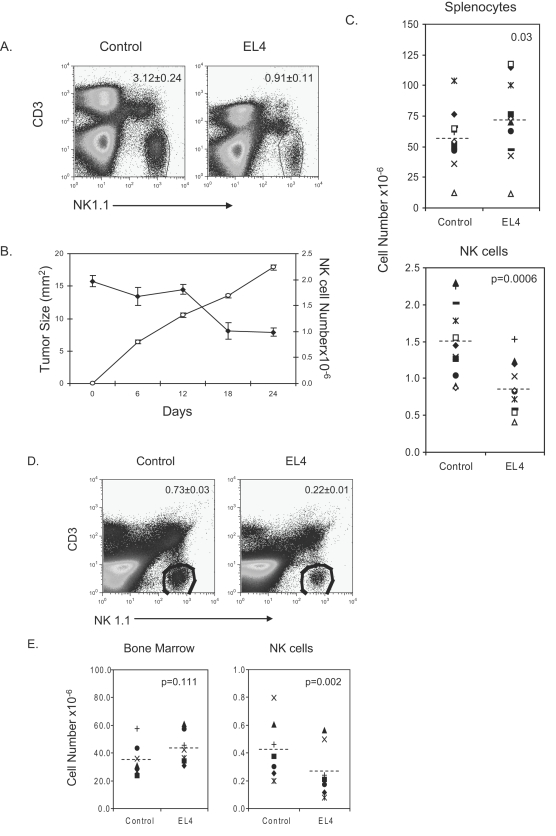
EL4 tumor growth causes a decrease in splenic and bone marrow NK cells. Mice were injected subcutaneously with 5×10^6^ EL4 cells and compared to mice that received PBS. The number of NK cells was calculated by multiplying the percentage of NK cells by the cellularity of the spleen or bone marrow. A. FACs profile of splenocytes stained with CD3 and NK1.1. NK cells were characterized as NK1.1^+^CD3^−^. The percentage of NK cells is given as the mean±SEM of five animals. B. Kinetics of tumor growth and reduction of NK cells. Spleens from EL4 bearing mice were obtained every six days and compared to control mice that were sacrificed on day 0. EL4 tumor growth was measured using calipers and the average diameter was obtained from the width and length of the tumor. C. Splenic cellularity and NK cell number from control and EL4-bearing mice. Each symbol represents the mean of a separate experiment. A paired T test was used to calculate the p-value. D. FACs profile of the bone marrow. NK cells are characterized as NK1.1^+^CD3^−^. The percentage of NK cells is given as the mean±SEM of five animals. E. Bone marrow cellularity and NK cell number obtained from control and EL4-bearing mice from several experiments. The number of NK cells was calculated by multiplying the percentage of NK cells by the number of bone marrow cells. Each symbol represents the mean of a separate experiment. A paired T test was used to calculate the p-value. Data in C–E were obtained when mice reached early removal criteria, i.e., the tumor reached 2 cm in diameters, usually at 4 weeks after tumor challenge.

Decreased numbers of NK cells have been observed in chronic mylogenous leukemia[17]. In order to study the effects of tumor cells on NK cell numbers, a suitable model was required. To determine if the transplantable tumor cell line, EL4, would diminish NK cell numbers a time course was performed to evaluate tumor size with NK cell numbers. EL4 tumor growth was monitored every 2–3 days, and every six days a subset of EL4 bearing mice were sacrificed to evaluate NK cell numbers. The reduction of splenic NK cells is exemplified by the FACs profiles in Fig 1A that shows a 3-fold reduction in NK cell percentage. The absolute number of NK cells also decreased as the tumor increased in size (Fig 1B). In multiple experiments comparing the cellularity of splenocytes obtained from EL4 bearing mice and the control mice, the EL4-bearing mice showed no reduction in splenocyte cellularity. However, the number of NK cells in the spleen was reduced (Fig 1C). Given evidence that NK cell development occurs in the murine bone marrow [19], [20] we evaluated the number of NK cells in that organ. As in the spleen, the percentage of NK cells was reduced in the bone marrow of tumor-bearing mice Fig 1D. With the cellularity in the bone marrow being equal, the reduced percentage of NK cells coincided with a reduction in NK cell numbers (Fig 1E.) These data demonstrate that EL4 reduces NK cell numbers.

To determine if a reduction of NK cells occurs with other murine tumors, C57Bl/6 mice were challenged with MC38 colon cancer and B16F1 melanoma. Mice bearing MC38 tumors had a strong reduction of NK cells in the bone marrow with a less significant drop off of NK cells in the spleen (Table 1). Different from EL4 and MC38 the B16F1 tumor demonstrated a greater reduction in NK cell number in the spleen than in the bone marrow (Table 1). These findings suggest that multiple tumors lead to a reduction in NK cell numbers in both the bone marrow and spleen.

**Table 1:** Multiple tumor cell lines reduce NK cell numbers.

Breed	Number of Mice	Number of Experiments	Tumor	Spleen	p-value	Bone Marrow	p-value
C57Bl/6	28	7	Control	1.63×10^6^±1.9×10^5^		4.27×10^5^±8.0×10^4^	
	28		EL4	1.01×10^6^±1.3×10^5^	0.005*	2.68×10^5^±7.1×10^4^	0.002*
	4	1	Control	1.42×10^6^±2.2×10^5^		1.01×10^6^±2.2×10^5^	
	4		B16F1	0.54×10^6^±1.6×10^5^	0.010	0.59×10^6^±1.2×10^5^	0.053
	9	2	Control	1.34×10^6^±7.4×10^5^		2.70×10^5^±1.8×10^4^	
	9		MC38	0.74×10^6^±0.2×10^5^	0.32*	0.96×10^5^±1.8×10^4^	0.001*

* p-value was calculated using a paired t test.

### NK cell Reduction is independent of apoptosis, proliferation and IL15

**Figure 2:**
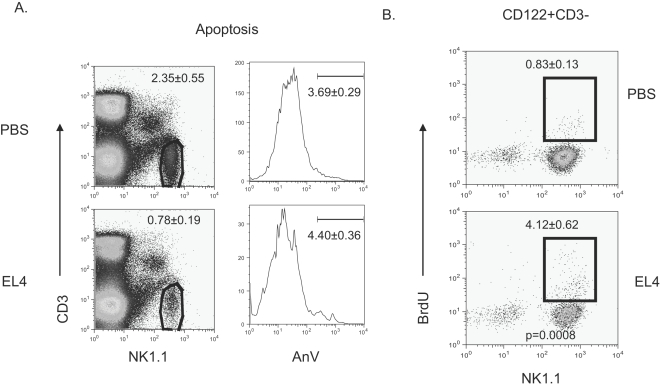
Apoptosis and NK cell proliferation do not account for NK cell depletion. Mice were injected subcutaneously with PBS or 5×10^6^ EL4 cells. After 3 weeks, control and EL4-bearing mice were sacrificed and splenocytes were stained. A. The percentage of Annexin V^+^NK1.1^+^CD3^−^ cells was used to determine the level of NK cell apoptosis. B. Three hours prior to euthanasia control and tumor-bearing mice were injected with BrdU. Splenic NK cells were then stained for intracellular BrdU. The splenic NK cell population was gated on CD122^+^CD3^−^ cells. Data shown were means and SEM, involving 5 mice per group.

Patients with head and neck cancer, breast cancer and chronic myelogenous leukemia have demonstrated reduced numbers of NK cells due to an increase in NK cell apoptosis[21], [22]. To determine if apoptosis causes a reduction in NK cells numbers in EL4-bearing mice, Annexin V staining was performed on splenic NK cells. As shown in Fig. 2A, a reduced percentage of NK cells was observed in the spleen of tumor-bearing mice. The percentage of NK cells undergoing apoptosis in these mice, however, was not significantly different than that found in the control mice. Bone marrow and liver cells were also stained for Annexin V, but again, no difference was found in the percentage of NK cells undergoing apoptosis (data not shown).

An alternative explanation for the reduction in NK cell numbers seen in tumor-bearing mice is that NK cell proliferation is reduced. To examine the proliferation of NK cells we pulsed tumor-free and tumor-bearing mice with BrdU three hours prior to euthanasia. Splenocytes were stained for the incorporation of BrdU. As shown in Fig. 2B, the percentage of BrdU positive cells was actually higher among NK cells in the spleen of tumor-bearing mice. There no defect in proliferation can account for the decrease in NK cell numbers.

**Figure 3:**
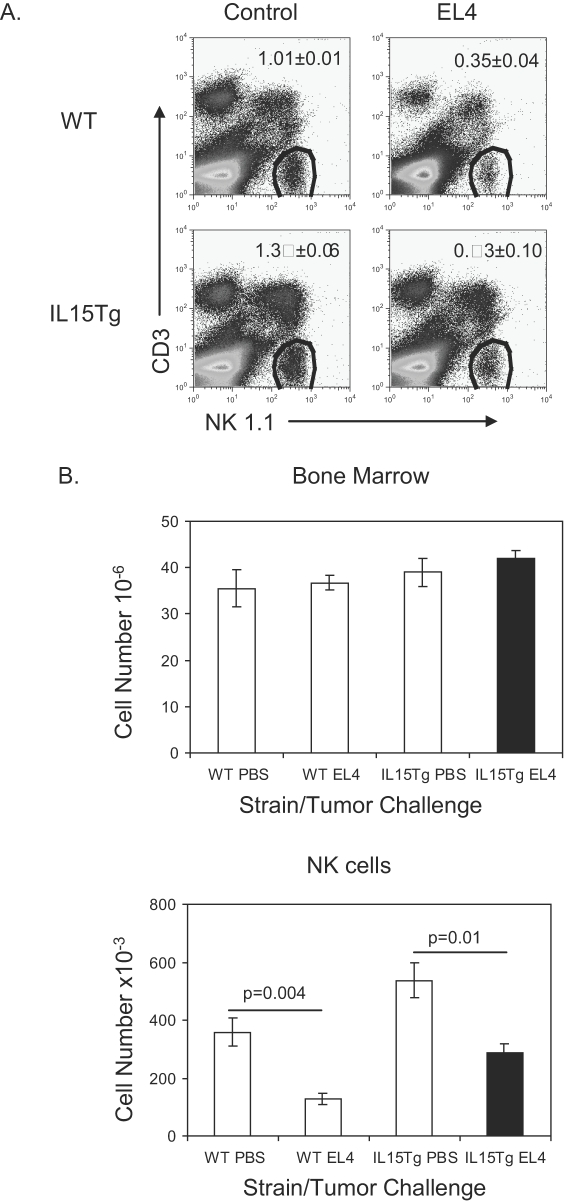
IL15 does not prevent loss of NK cell number. IL15Tg and littermate controls were injected with PBS or EL4. After 3 weeks, bone marrow was evaluated for NK cell number. A. FACs profile of NK1.1^+^CD3^−^ cells obtained from WT or IL15Tg mice with or without tumor. B. Bone marrow cellularity and absolute number of NK cells. The absolute number of NK cells was calculated by multiplying the percentage of NK1.1^+^CD3^−^ cells by the number of bone marrow cells. Data shown were means and SEM, involving 4 mice per group.

**Figure 4:**
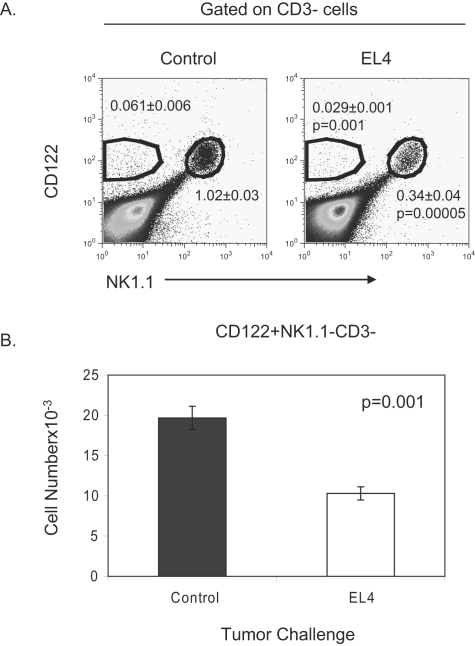
NK cell progenitors are reduced in EL4 tumor-bearing mice. B6 mice were injected with PBS or EL4 and sacrificed after 21 days. NK cell progenitors were characterized as CD122^+^NK1.1^−^CD3^−^. A. FACs profile of NK cell progenitors. B. Absolute number of progenitors was calculated by multiplying the percentage of CD122^+^NK1.1^−^CD3^−^ cells by the total number of NK cells. Data shown were means and SEM, involving 4 mice per group.

Interleukin-15 knock out mice have reduced numbers of NK cells[8]. To determine if IL15 deficiency perpetuated the loss of NK cells seen in tumor-bearing mice, mice that over express IL15 were challenged with EL4 to determine if IL15 prevents a loss in NK cell number. PBS and EL4 were injected into IL-15Tg mice or littermate controls. As expected IL-15Tg mice displayed an increased percentage of NK cells compared to wild type control mice (Fig 3A). However, like wild type control mice, the presence of EL4 in IL-15Tg mice resulted in a reduction in the percentage of bone marrow NK cells (Fig 3A). The cellularity in the bone marrow of EL4-bearing, IL-15Tg mice was the same as IL-15Tg control mice and wild type mice (Fig 3B). Thus, IL-15Tg and wild type control mice that have EL4 tumors have decreased NK cell numbers (Fig 4B), and this decrease in NK cell numbers is independent of IL15.

### Tumor-associated decrease in common lymphoid progenitors

In vitro studies have demonstrated that IL15 does not induce the generation of NK cells directly from hematopoietic stem cells [23]. Instead a cocktail of cytokines that include Flt3L, KitL, IL7 and IL6 are required to induce IL-15 responsiveness, after which, the addition of IL-15 will result in the generation of NK cells [23]. The acquisition of CD122 is thought to commit cells to the NK cell lineage and IL-15 responsiveness [24]. To determine if there is a defect in NK cell precursors (NKP) we injected mice with EL4 and PBS. NK cell progenitors were initially characterized as CD122^+^NK1.1^−^CD3^−^. FACs analysis of the bone marrow showed a reduction in the NK1.1^+^CD3^−^ population, which demonstrates a decrease in NK cells (Fig 4A). Furthermore, the CD122^+^NK1.1^−^CD3^−^ population also decreased suggesting a decrease in NKP (Fig 4A). Upon calculating the absolute number of NKP, there was an approximate 2-fold reduction in tumor-bearing mice (Fig 4B). These data suggest a defect in the acquisition of NKP as a cause for reduced NK cells in EL4 bearing mice.

**Figure 5:**
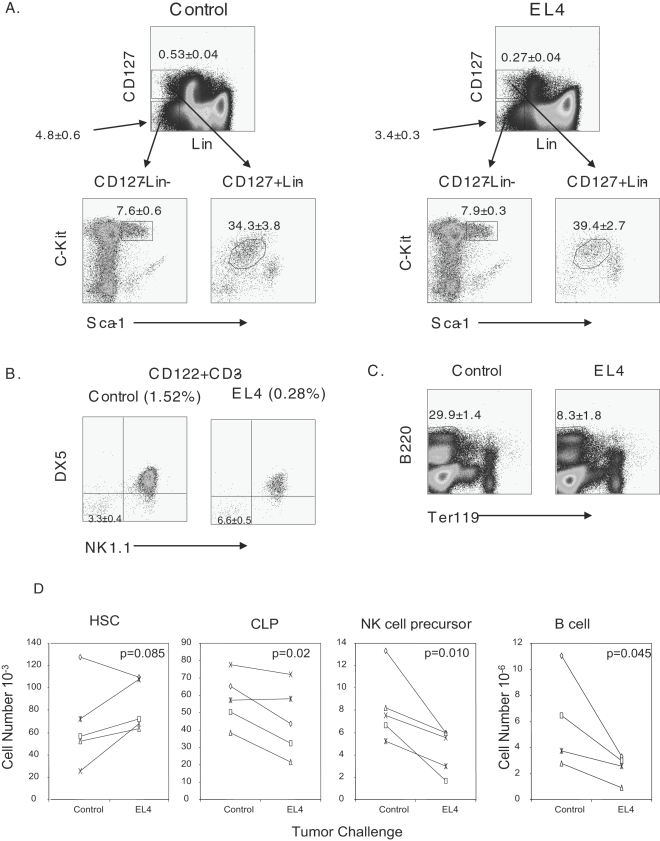
Altered lymphopoiesis. B6 mice were injected with PBS or EL4 and sacrificed after 21 days. A. FACs profile of early hematopoiesis. HSCs are characterized as Lin^−^CD127^−^Sca1^+^cKit^+^ while the CLP is characterized as Lin-CD127^+^Sca1^+^cKit^+^. B. Characterization of NKPs as CD3^−^CD122^+^NK1.1^−^DX5^−^. C. Characterization of B cells by B220. D. Absolute numbers of HSCs, CLPs, NKPs, and B cells. Each line and symbol represents the mean of separate experiments involving 5 mice per group. A paired T test (one-tail) was used to determine whether tumor growth reduced the number of HSC, CLP, NKP and B cells. FACS data shown in A–C are representative of those obtained from 5 independent experiments, involving a total of 5 mice per group.

Given the plausibility that NK cell progenitors are decreased in tumor bearing mice, there may be further hematopoietic defects. To evaluate this hypothesis, tumor-bearing mice were evaluated with a panel of antibodies to evaluate the number of NK cells, NK cell progenitors, common lymphoid progenitors (CLP), hematopoietic stem cells (HSCs) and B cells. HSCs belong to the Lin^−^CD127^−^cKit^+^Sca1^+^ population[25]. To evaluate the HSC pool, gated Lin^−^CD127^−^ cells were evaluated for cKit^+^Sca1^+^ (Fig. 5A). The percentage of HSCs from both EL4-bearing mice and control mice were approximately equal, suggesting that the HSC pool was not affected by tumor growth. The CLP, characterized as Lin^−^CD127^+^cKit^int^Sca1^int^ [26], however, was reduced by about 50% (Fig 5A). The major point of reduction was associated with a decrease in CD127 expression as the corresponding cells that expressed intermediate levels of cKit and Sca1 were approximately equal (Fig 5A). To more stringently define NKP, bone marrow cells were stained for the NKP as marked by CD122^+^NK1.1^−^DX5^−^CD3^−^ [24]. Bone marrow cells were gated on the CD122^+^CD3^−^ cell population, which were greatly reduced in EL4-bearing animals (Data not shown). The gated population was then evaluated for NK1.1 and DX5 expression. Interestingly, there was a slight increase in the percentage of CD122^+^NK1.1^−^DX5^−^CD3^−^ (Fig. 5B), although the absolute number was decreased. Since NK cells are a third subset of lymphocytes, we tested whether B cell number is also reduced, as these cells also develop in the bone marrow. Interestingly, we also observed a significant decrease in the percentage of B cells as characterized by the B cell marker B220 (Fig 5C). The absolute number of cells correlated well with the percent reduction is observed in Fig 5A–C. In several experiments HSCs were relatively equal, while there were decreases observed in the CLPs, NKPs and B cells (Fig 5D). These data suggest that lymphopoiesis is altered in EL4-bearing animals.

**Figure 6:**
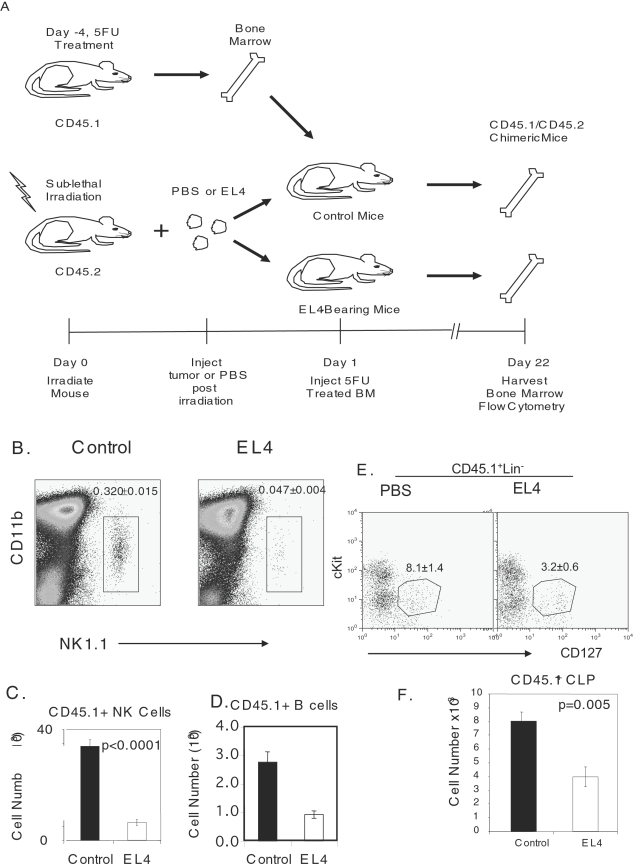
Decrease in CLPs, NKPS and Block in NKP progression. B6 mice were sublethally irradiated with 500 Rad prior to EL4 or PBS injection. Twenty-four hours later 5FU treated bone marrow from congenic CD45.1 mice was injected into control or EL4-bearing mice. Three weeks later bone marrow was harvested to evaluate NK cell progenitors. Donor NK cells were identified by an anti-CD45.1. A. Diagram of experiments. B. FACs profile of CD45.1^+^ NK1.1^+^ cells. Data shown are from gated CD45.1^+^CD3^−^ cells. The numbers shown in the panels are means and SEM, involving a total of 5 mice per group. C. Absolute number of CD45.1^+^ NK cells. D. Absolute number of donor B cells. E. FACS profile of donor CLP, characterized as CD45.1^+^Lin^−^CD127^+^cKit^int^. Data are representative of 5 mice per group. F. Absolute number of CD45.1^+^ and CD45.1^−^ CLP. Data shown are means and SEM, involving a total of 5 mice per group.

To confirm that there is an alteration in lymphopoiesis in EL4-bearing animals a bone marrow transplant was performed. Mice were sub-lethally irradiated prior to tumor injection. One day later, 5FU treated bone marrow cells, from CD45.1 congenic mice were injected intravenously into control and tumor bearing mice and cellular differentiation was evaluated approximately three weeks later. In general, it was found that CD45.1 cells were expanded to a greater extent in tumor bearing mice (data not shown). However, the donor NK cell population was dramatically decreased (Fig 6B). Absolute numbers of donor NK cells (Fig. 6C) and B cells (Fig 6D) were also decreased.

Since NK and B cells are both derived from CLP, we tested the hypothesis that a reduction in this subset is an underlying cause for both defects. As shown in Fig. 6E and F, the donor CLP, as defined by CD45.1^+^Lin^−^CD127^+^cKit^int^ markers, was reduced in both % and absolute numbers. Taken together, the congenic transfer of CD45.1^+^ cells into control and tumor bearing mice demonstrate that tumor growth leads to a block of differentiation into the lymphoid lineage.

## Discussion

In a previous study we demonstrated that EL4 tumors secrete a soluble factor that modifies IL15Rα expression, which is associated with defective NK cell differentiation[6]. One aspect of NK cell development that was not thoroughly evaluated in that manuscript was the decrease in NK cells found in EL4-bearing mice. As we found that IL15Rα was decreased in the bone marrow, we hypothesized that modifications of hematopoietic processes may result in a decrease in NK cell number.

Hematopoiesis is a tightly regulated process. Steady state hematopoiesis is regulated in the bone marrow by cytokines that act in a paracrine manner to maintain the production of all the different hematopoietic lineages[3]. It is only upon a stress in which cytokines released into circulation act in an endocrine manner to modify hematopoietic production[3]. Tumors are a prime example of a stress that results in the production of cytokines. Many transplantable tumors have been found to secrete cytokines that modify hematopoeisis[4]. Some of these cytokines include TGF-β, VEGF-A and GM-CSF amongst others[27], [28]. The ability for tumor growth depends on their ability to obtain nutrients from the circulation. To meet this demand many tumors secrete VEGF-A, which induces vasculariztion[29]. VEGF-A has been shown to act on bone marrow cells, which leads to an increase in GR-1/CD11b immature myeloid cells[5].

In EL4-bearing mice, GR-1/CD11b myeloid cells were increased in both the bone marrow and spleen (Data not shown). The development of GR1/CD11b myeloid cells is associated with a large tumor burden and a state of immune suppression[30]. Immune suppression by GR1/CD11b is associated with a decrease in T cell function[30]. Interestingly, ablation of NK cells in BW-Sp3 tumor-bearing mice results in favorable growth conditions for GR-1/CD11b myeloid cells[31]. In Figure 1B we observed an inverse relationship between NK cell number and tumor growth.

There have been several reports that have demonstrated a decrease in NK cell number in patients with tumors[17], [21], [22]. Most of these reports have shown that there was an increase in NK cell apoptosis. In evaluating NK cell apoptosis we observed no significant difference in cellular death. Furthermore, there was an increase in NK cell proliferation. Lymphopenic animals undergo homeostatic proliferation to restore T cell numbers and a similar phenomenon may be occurring within the NK cell compartment of tumor-bearing mice in an attempt to restore the number of NK cells [32]. With equal levels of cellular death and increased proliferation of NK cells in EL4 bearing-mice compared to control mice there should be a net increase in the NK cell number. However, we demonstrated that the pool of NK cells continually drops with tumor enlargement.

Two independent studies have demonstrated that transgenic expression of Bcl2 maintains NK cell number in IL15^−/−^ mice and that IL15 is important for NK cell survival[15], [16]. Early hematopoietic progenitor cells are not responsive to IL15 and upon upregulation of CD122 hematopoietic precursors become commited to the NK cell lineage[23], [24]. However, IL-15 transgene failed to rescue decreased levels of NK cells after tumor challenge. These data suggested that an earlier stage of NK cell development may be responsible for the decrease in NK cell numbers. The first candidate to consider was the NKP characterized as being CD122^+^NK1.1^−^DX5^−^CD3^−^ [24]. We observed two main differences in NKPs. First, the overall number of NKPs was reduced in tumor-bearing mice compared to wildtype animals. The reduction was similar to that seen in total NK cell numbers. Therefore the decrease in NK cell number is likely due to reduction in the number of cells entering into the NK cell lineage. Second, when NKPs were gated on CD122^+^CD3^−^ cells there was an approximate two-fold increase in the percentage of NK1.1^−^DX5^−^ cells in tumor-bearing mice compared to control mice. This suggests that NKPs may also be blocked in their progression to become NK cells. To exemplify this point it was observed in congenic bone marrow transplants that there was a 2 to 3 fold accumulation of recipient and donor NKPs, in tumor-bearing mice compared to control mice.

Given that NK cells are a third subset of lymphocytes, it is plausible that earlier progenitor cells are also impaired in tumor-bearing animals. Our data revealed that a reduction in NK cells in tumor-bearing animals also correlates with a decline in the number of CLP. As with NK cells and NKPs, the CLP is reduced by approximately 50% in tumor-bearing animals while the HSC population is constant. Furthermore, there was a substantial decrease in B cell development. Defects in B cell development have previously been reported in transgenic mice that overexpress arginase[33]. Increases in arginase concentration have been associated with the production GR-1/CD11b myeloid cells[4].

The mechanism by which NK cells are affected by tumors remains elusive. We have evaluated antibodies to neutralize TGF-β in the EL4 model and blocking antibodies against VEGFR1 and VEGFR2 in the MC38 model (Data not shown). Neutralization of TGF-β, TNFα and blocking antibodies against VEGFR1 and VEGFR2 had no effect on NK cell number (data not shown). We also evaluated EL4 transcripts for GM-CSF, G-CSF, SCF and IL6 amongst others in an RNAse protection assay to determine if any cytokines that potentially inhibit NK cell development could be detected (data not shown). There was no indication of cytokines that modify hematopoietic development in EL4.

Regardless of the mechanism, our study appears to be the first to show tumor growth leads to reduced lymphopoiesis, which in turn results in a decrease in NK and B cell numbers. Linking tumor growth to abnormal hematopoiesis in animal model may lead to new approaches aiming at boosting host immunity to cancer.
